# Driving Outcomes among Older Adults: A Systematic Review on Racial and Ethnic Differences over 20 Years

**DOI:** 10.3390/geriatrics3010012

**Published:** 2018-03-20

**Authors:** Ganesh M. Babulal, Monique M. Williams, Sarah H. Stout, Catherine M. Roe

**Affiliations:** 1 Charles F. and Joanne Knight Alzheimer’s Disease Research Center, Washington University School of Medicine, St. Louis, MO 63108, USA; shstout@wustl.edu (S.H.S.); cathyr@wustl.edu (C.M.R.); 2 Department of Neurology, Washington University School of Medicine, St. Louis, MO 63110, USA; 3 VITAS Healthcare, St. Louis, MO 63146, USA; Monique.Williams@vitas.com

**Keywords:** race, ethnicity, disparities, driving, older adults, health

## Abstract

The population of older adults (aged 65 years and older) in the United States will become more racially and ethnically diverse in the next three decades. Additionally, the growth of the aging population will come with an expansion in the number of older drivers and an increased prevalence of chronic neurological conditions. A major gap in the aging literature is an almost exclusive focus on homogenous, non-Hispanic white samples of older adults. It is unclear if this extends to the driving literature. A systematic review of SCOPUS, PubMed, CINAHL Plus, and Web of Science examined articles on driving and racial/ethnic differences among older adults. Eighteen studies met inclusion criteria and their results indicate that racial and ethnic minorities face a greater risk for driving reduction, mobility restriction, and driving cessation. The majority of studies compared African Americans to non-Hispanic whites but only examined race as a covariate. Only four studies explicitly examined racial/ethnic differences. Future research in aging and driving research needs to be more inclusive and actively involve different racial/ethnic groups in study design and analysis.

## 1. Introduction

People are living and driving longer than ever before. By 2050, an anticipated doubling of the population of older adults (84 million) will be accompanied by more older motorists (25% of all drivers) in the United States alone [1,2]. Driving is crucial for access to services and social participation, making it a cornerstone supporting identity and independence [3]. Conversely, studies have shown that advanced age is associated with functional impairments, greater difficulty with maintaining driving skills, and a higher risk of motor vehicle crashes [4]. Given these important issues, research efforts continue to identify risk factors to assess driving decline and safety among adults 65 years of age and older [5,6].

Additionally, over the next four decades, the population of older adults will be more racially and ethnically diverse in the United States. By 2060, the older adult population of African Americans is expected to increase to 12% (from 9%), Hispanics will increase to 22% (from 8%), and Asians will increase to 9% (from 4%), while non-Hispanic whites will decrease to 55% (from 78%) [7]. Compared to non-Hispanic whites, both African American and Hispanic older adults have a higher prevalence of chronic diseases, including neurological conditions, and greater mortality. They also continue to disproportionally experience health disparities with higher costs associated with hospital and long-term care [2,8].

Ironically, however, the current research landscape in aging and health outcomes predominantly gains new knowledge using samples of non-Hispanic whites (i.e., with limited representation of minority groups) [9,10]. There are numerous reasons for this gap in research, including recruitment/enrollment bias, mistrust of research, racism, lack of represented staff, language, and cultural barriers [11]. It is unclear whether the research on racial and ethnic minorities and driving behaviors among older adults is plagued by similar problems found in the greater aging literature. Driving outcomes may include cessation, crash risk, safety, and decline in performance. These outcomes contribute to one’s ability to age in place and remain independent while supporting well-being and quality of life. The purpose of this systematic review is to examine the body of literature on driving outcomes among older adults and to determine whether minority and ethnic drivers are underrepresented in the sample size, whether the existing literature suggests that there are any racial or ethnic differences in driving, and if so, to explore the associated implications for those minority populations.

## 2. Materials and Methods

### 2.1. PICOS Framework

We employed the PICOS (Population, Intervention, Comparison, Outcome, Study Design) approach to determine the structure and scope of this systematic review. Our population/participants of interest included older adult drivers, which was operationally defined as those aged 65 years or older. Use of interventions was not a focus of this review; however, if an intervention was used, it was examined in the context of driving. We sought out studies that used control/comparator groups to evaluate driving outcomes between two or more racial/ethnic groups. The outcome of interest could vary but was required to be contextually relevant to driving mobility and could include driving decline, driving performance, crash risk, and/or driving cessation. Finally, study design was not restrictive to any particular design and could include randomized control trials, prospective, retrospective, cross-sectional, or longitudinal designs.

### 2.2. Literature Search

Given the preponderance of research studies on older adults, and health disparities, a specific search strategy based on the aforementioned PICOS criteria was used to identify studies that directly examined driving outcomes among racial or ethnic minorities. The SCOPUS, PubMed, CINAHL Plus (EBSCO), and Web of Science databases were searched using specific search terms. These terms were “racial disparity AND driving”, “race AND driving AND/OR older adult”, “ethnicity AND driving AND/OR older adult”, and “minority OR race AND driving”. These search terms were intended to be inclusive of the literature spanning, older adults, any form of driving mobility, and race/ethnicity.

### 2.3. Inclusion and Exclusion Criteria

Titles and abstracts were screened and excluded on the following operationalized criteria: (1) published before 1997 (e.g., more than 20 years old), (2) no exclusive focus on older adults (age ≥ 65 years), (3) driving mobility as an outcome was not examined (e.g., decline, performance, crash risk, and/or driving cessation), (4) not written in the English language, (5) article’s study design did not fit those identified in the PICOS approach (e.g., commentary, book chapter, or non-peer-reviewed paper).

### 2.4. Data Extraction, Assessment, and Qualitative Synthesis

Queried article citations (titles and abstracts) across the four databases were downloaded into Endnote X8 reference manager. A separate library was created for each respective database. Articles in each library were initially screened and duplicate publications with the same title but over consecutive years were removed. Next, all article citations were screened according to the aforementioned five exclusion criteria. If information was missing from the abstract required to make a sufficient determination, the full text article was obtained to determine relevance and inclusion/exclusion in the review. Citations remaining after this initial screen of title and abstract were reviewed in-depth for relevance to the purpose of the systematic review. A full text article was obtained and reviewed again based on the inclusion/exclusion criteria. Articles with a focus on older adults and driving and that reported or focused on race/ethnicity were included in this review. Given that driving as an adjective and verb can be used in a variety of lexical conditions and contexts, if driving was not discussed as an activity in the context of operating a vehicle, the article was excluded. Driving-related outcomes like crashes, decline, performance, and cessation are contextually relevant to older adults. Each article was reviewed to identify the study’s purpose and design, age of participants, sample size, the racial/ethnic makeup of the sample, whether there was an explicit focus on examining racial/ethnic differences, and if any statistically significant differences were found with respect to driving mobility and associated outcomes. This information was consolidated and presented in a table along with a discussion of sample size make up and associated implications for minority populations with respect to driving mobility.

## 3. Results

**Figure 1:**
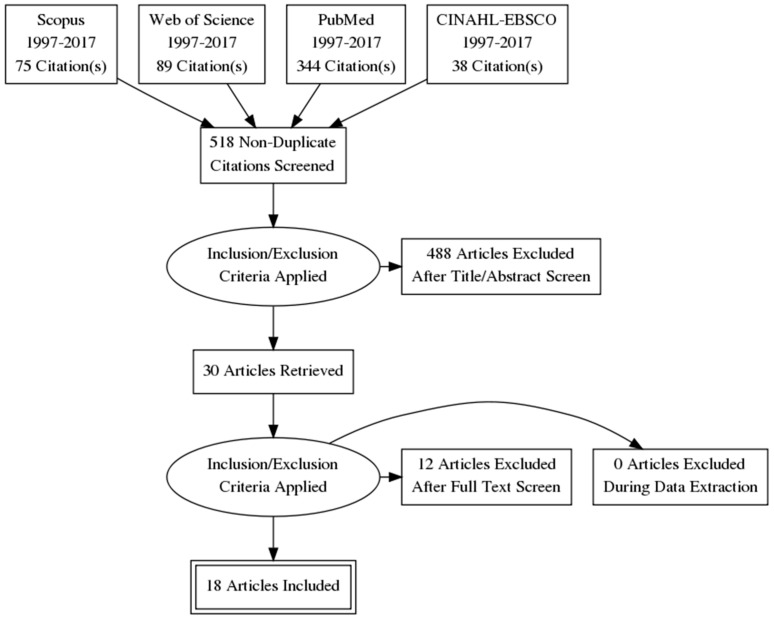
PRISMA flow diagram of publications included in systematic review.

The search across all four databases yielded 546 articles (Figure 1). There were 28 duplicate articles which were immediately excluded, resulting in 518 publications for screening. After the application of the inclusion/exclusion criteria and initial screen of the title and abstract, 30 articles remained. Of the 488 articles, 41 were published before 1997, 89 did not exclusively focus on older adults (age ≥ 65 years), and the remaining articles did not focus on driving as an activity or outcome. Each article was then thoroughly reviewed for its focus on older adults, race/ethnicity, and driving outcomes. Following this review, an additional 12 articles were excluded due to the inclusion of younger age groups (*n* = 4), being a qualitative study (*n* = 2), or failure to examine driving as an activity as the main outcome (*n* = 6). In the remaining articles, we assessed the study design, purpose/objective, age range, total sample size, and inclusion of racial/ethnic group in the sample size, and then identified if there was an explicit focus on race/ethnicity, and finally whether there were any group differences found.

The 18 publications spanned 19 years (Table 1); seven used a prospective longitudinal design, five used a retrospective longitudinal design, one was cross-sectional, four used data from a randomized control trial (RCT), and one used a cross-sectional case-control design [12,13,14,15,16,17,18,19,20,21,22,23,24,25,26,27,28,29]. Ten studies examined risk factors associated with driving and crashes, five sought to characterize differences in driving behavior, and three investigated driving cessation among older adults. The majority of studies used samples of older adults, aged 65 years or older, with only three studies including adults aged 55 years and older. Sample size ranged from 120 to 17,349, where larger samples used data from national studies like the Health and Retirement Study (HRS) or an RCT such as the Advanced Cognitive Training for Independent and Vital Elderly (ACTIVE) study [15,16,18].

In addition to non-Hispanic whites, nine studies included African Americans specifically, six studies lumped different racial/ethnic groups into “other” or a “mixed” category, two studies examined racial groups including African Americans and other, and one study included African Americans, Hispanics, and other as groups. Fourteen of the studies had racial/ethnic groups represented in <20% of the total samples. Only four of the 18 studies had an explicit focus on race/ethnicity, while the remaining 14 studies treated race as a covariate in their analyses [15,16,17,18]. Additionally, the four studies that had an explicit focus on race/ethnicity had larger sample sizes (>2700) and the representation of racial/ethnic minorities was more than 20% of the total sample. Finally, only six of the 18 studies found a statistically significant difference in the outcomes, with all four studies that explicitly examined racial/ethnic differences finding some significant differences.

In one of the six studies reporting racial/ethnic differences (Table 1), using a small sample of older adults, Sims and colleagues (1998) examined associations between self-reported medical and functional outcomes and police-reported crashes; African American race was related to a higher likelihood of involvement in a crash [29]. However, African Americans only accounted for 14.9% of the total sample. Choi and colleagues used data from the ACTIVE study to examine several driving outcomes among older adults using large sample sizes [14,15,16,17]. They found that racial minorities were at a greater risk of driving cessation in later life than non-Hispanic whites [16]. Compared to non-Hispanic whites, Choi and colleagues (2015) found that African Americans experienced more life-space constriction (limited mobility in geographic and spatial areas) at baseline, but non-Hispanic whites experienced more life-space constriction over a five-year period [17]. Using data from the HRS, Choi and Mezuk found that women who were ethnic minorities were more likely to have never driven and have less wealth and education compared to former drivers [15]. Using a sample of over 17,000 older adults (≥65 years) from the HRS, Dugan and Lee found that non-Hispanic white older drivers were more likely to exhibit current and future safe-driving behaviors compared to minority races [18]. These findings validated earlier results from the ACTIVE study, also examined by Choi and colleagues [16]. Finally, Edwards et al. (2017) performed secondary data analysis using data from the Staying Keen in Later Life study to examine associations between hearing impairment and driving mobility and found that minority race was associated with restricted baseline mobility [19]. With the exception of the study by Sims et al., the remaining five studies all used self-report via questionnaires or interviews. Taken together, the results suggest that being part of a minority racial or ethnic group was associated with a greater risk for current and future driving cessation, crashes, mobility restriction, and life-space constriction [15,16,17,18,19,29].

## 4. Discussion

This systematic review examined the literature on older adults and driving to identify studies published to date that included racial and ethnic groups, to determine if any racial/ethnic differences in outcomes exist and if there were associated implications. We reviewed publications over the past two decades across four major databases and found 18 studies that fit the inclusion/criteria. Studies ranged across 19 years, with significant variation in the types of study design, sample size, age range, driving outcome, and percentage of racial/ethnic groups represented in the total sample. A majority of the studies simply treated race/ethnicity as a covariate in the analyses and did not find any group difference. Data from the few studies that had an explicit focus on race/ethnicity indicate that health disparities in driving behavior and outcomes exist between non-Hispanic whites and racial and ethnic minorities in the US. The results from this handful of studies suggest that racial/ethnic groups may have a higher risk of driving cessation, mobility limitations, and life-space restriction.

This line of investigation in driving outcome is timely given the limited past research and the projected growth of racial and ethnic groups and older adults in the next few decades in the United States. Driving will continue to be a valued mode of transportation, not only because of its utility but also because it provides a sense of independence, meaning, and choice. While public transportation may be readily available, most older drivers prefer transportation in a personal vehicle [3]. These findings are important because driving cessation and mobility limitations are linked to higher rates of depression, faster time to institutional care (e.g., assisted living, skilled nursing facilities), poorer functional outcomes, and a greater risk of mortality [4]. The impact of cessation is further emphasized since both older men and women can expect to spend seven to 10 years as a non-driver at the end of their life, being reliant upon other modes of transportation [30]. The anticipated increase in older adults warrants studies identifying important risk factors that compromise driving outcomes.

The result of this systematic review sheds light on a number of limitations present in the extant driving and aging literature. Only four studies explicitly examined racial/ethnic differences among older adults and driving. Studies that may have examined racial/ethnic differences, but did not find any statistically significant differences and did not report it in the publication, would not have been captured in the search strategy. While African Americans are represented in a majority of the studies, the overall percentages and number of persons in the total sample was low (<20%), resulting in potential biases associated with study design, including, selection, generalizability, adequate power, and reproducibility [16,31,32]. Furthermore, many racial/ethnic groups tend to be lumped together and labeled as ‘mixed’ or ‘other’ in the analyses. This form of data reduction, while helpful for conducting analyses for groups with smaller numbers, also undermines the examination of key differences related to diversity that may be present when studying driving differences in majority and minority racial and ethnic groups. Driving behavior was largely explored via self-report or review of data from law enforcement and departments of motor vehicles. The limitations of self-report data include social desirability bias, misinterpretation, memory recall, and fixed choices on questionnaires [18,33,34]. Driving performance was examined in one study on a standard road test [13], but these results should be interpreted with caution since driving skills tend to be overlearned [35,36].

Prior studies on driving have explored and successfully used naturalistic methodologies to understand driving behaviors in older adults [36,37,38]. Future studies should examine older adult cohorts with neurological disorders such as mild cognitive impairment or dementia, and employ the use of naturalistic driving methodologies that can monitor driving behavior in real time, along with self-reported driving behaviors. Prospective longitudinal cohorts of non-Hispanic whites and different ethnic and racial groups of cognitively normal older adults can help to address individual differences in driving outcomes, including, decline and eventual cessation. These studies may also help to address the early identification of older adults at risk of crashes and driving cessation. To date, studies on health disparities examining racial and ethnic minorities, driving, and neurological disease are very limited. This problem centers on a lack of overlap between these three independent bodies of literature: a neurological disease, driving, and race. Examining driving among older adults through a health disparities lens highlights this issue and elucidates the fact that racial and ethnic minorities may, unfortunately, not be considered a priority population for these studies.

Given the projected growth and diversity of the older adult population, there is a crucial need to better understand how the general population is impacted by issues surrounding driving decline, safety, and cessation. Part of this need should serve to examine racial and ethnic differences in driving given the numerous health disparities that are already known to impact minority populations, and that may have an even stronger effect on minority elders. It important for new and effective interventions or public policy to be based upon data generated from heterogeneous samples of diverse older adults. Part of the issue with a lack of inclusion of more diverse samples may be a result of a number of challenges encountered in the recruitment of racial and ethnic minorities [31,39]. Research into the recruitment and enrollment of different groups into scientific research has engendered a number of effective strategies to mitigate these challenges [11,40,41,42]. Other barriers affecting participation in driving studies may include access, ownership, and maintenance of a vehicle, exclusive use of public transportation, time availability to commit to research, or a lack of interest. These lifestyle factors should also be considered at the onset of study design. It is incumbent upon researchers to partner with community leaders and minority groups to focus on issues of health disparities and inclusion of diverse populations at the onset of their research. As evidenced by only a handful of existing publications [15,16,17,18] from this systematic review, more informed research is needed. Future studies will need to examine questions about whether older adults from racial/ethnic minorities (1) are at a higher risk of being involved in crashes among representative samples, (2) experience earlier changes in driving behavior compared to their non-Hispanic white counterparts, (3) show differences in adverse behaviors like speeding or hard braking, and if so, whether these behaviors are predictive of future decline, (4) have health disparity effects from early life that extend the ability to age in place, and (5) have different attitudes and expectations toward aging and driving in later life compared to non-Hispanic whites.

## 5. Conclusions

Health disparities are more prevalent in the aging population and may become compounded for racial and ethnic minorities. Based on this systematic review, health disparities are also found in driving, such that older adults from racial and ethnic minority groups have an increased risk for driving reduction, mobility restriction, and driving cessation compared to non-Hispanic whites. The existing studies found in this systematic review generally focus on African Americans and tend to group other minorities into “other” or “mixed” categories. Most of the studies identified had disproportionate samples of racial/ethnic groups and used self-report driving data. Future research is needed to determine the specific impact of driving decline and safety on the ability to age in place and quality of life by including older adults from racially and ethnically diverse groups in research samples, by examining the role of neurological diseases, and by using methodologically comprehensive driving outcomes.

## Figures and Tables

**Table 1:** Characteristics of publications included in systematic review.

	First Author (Year)	Study Design	Purpose	Age Range or Mean (SD) of *n* (in Years)	Sample Size	Racial/Ethnic Group *n* (% of Total Sample)	Explicit Focus on Race or Ethnicity	Group Difference Found in Driving Outcome
1	Edwards (2017) [18]	Retrospective Longitudinal	Impact of hearing impairment on driving mobility	63–90	500	African American 57 (11.4%)	No—Race/Ethnicity treated as covariate	Minority race associated with baseline-restricted mobility
2	Carr (2016) [12]	Prospective Longitudinal	Examine functional impairments and comorbidities on driving performance	64.9–88.2	129	African American 12 (9.3%)	No—Race/Ethnicity treated as covariate	No
3	Choi (2015) [16]	Randomized Controlled Trial	Examine gender and racial disparities in life-space constriction in later life	73.6 (5.9)	2765	African American 726 (26.2%)	Yes	African Americans have more life-space constriction at baseline but are stable over time
4	Choi (2014) [13]	Retrospective Longitudinal	Association between driving status and cognitive functioning in later life	71.9 (4.4)	9135	Mixed 1251 (13.6%)	No—Race/Ethnicity treated as covariate	No
5	Choi (2013) [14]	Retrospective Longitudinal	Characterize former vs. never drivers over 15 years	77.4 (4.44) and 77.4 (4.77)	3098	African Americans 539 (17.3%), Hispanics 327 (10.5%), Other 77 (2.4%)	Yes	Minority race was significant predicted to have never driven
6	Dugan (2013) [17]	Retrospective Longitudinal	Biopsychosocial risk factors associated with driving cessation	75.10 (7.16)	17,349	Mixed 3643 (20.9%)	Yes	Minority race a risk factor for current and future driving cessation
7	Green (2013) [20]	Retrospective Longitudinal	Examine sensory impairment as risk factor for crashes among older drives	70–99	1998	African American 350 (17.5%), Other 9 (<1%)	No—Race/Ethnicity treated as covariate	No
8	Choi (2012) [15]	Randomized Controlled Trial	Examine gender and racial disparities in driving cessation	73.54 (5.88)	2645	African American 394 (14.9%), Other 17 (0.06%)	Yes	Minority race more likely to stop driving faster in later life
9	Ball (2010) [11]	Randomized Controlled Trial	Examine the effect of cognitive training on subsequent crashes among older adults	65–91	908	African American 164 (18.0%)	No—Race/Ethnicity treated as covariate	No
10	Munro (2010) [23]	Cross-sectional	Examine risk factors that predict lane-changing errors in older adults	67–87	1080	African American 129 (11.9%)	No—Race/Ethnicity treated as covariate	No
11	Edwards (2009) [19]	Prospective Longitudinal	Examine driving status as a predictor of mortality among older adults	73.16 (2.77)	660	African American 94 (14.2%)	No—Race/Ethnicity treated as covariate	No
12	Lunsman (2008) [21]	Randomized Controlled Trial	Examine what factors predict change in visual processing	65–94	690	African American 185 (26.8%), Other 9 (1.3%)	No—Race/Ethnicity treated as covariate	No
13	Okonkwo (2008) [24]	Prospective Longitudinal	Examine self-regulation of older adults via driving habits and visual attention	75–100.44	1543	Other 41 (2.6%)	No—Race/Ethnicity treated as covariate	No
14	Owsley (2002) [26]	Prospective Longitudinal	Examine cataract surgery as a risk factor for crashes among older adults	71.2 (6.6) and 71.5 (5.4)	277	Other 37 (13.3%)	No—Race/Ethnicity treated as covariate	No
15	MacGregor (2001) [22]	Prospective Longitudinal	Examine if traffic sign test can distinguish older adult driver who crashed	65–91	120	Other 21 (17.5%)	No—Race/Ethnicity treated as covariate	No
16	Sims (2000) [27]	Prospective Longitudinal	Identify medical and functional risk factors for at-fault crashes	57–91	174	African American 26 (14.9%)	No—Race/Ethnicity treated as covariate	No
17	Sims (1998) [28]	Cross-sectional Case-control	Identify medical and functional risk factors for at-fault crashes	57–91	174	African American 26 (14.9%)	No—Race/Ethnicity treated as covariate	African American race was associated with more at-fault crashes
18	Owsley (1998) [25]	Prospective Longitudinal	Examine visual processing impairment as a risk factor for crashes	55–87	294	African American 56 (19%)	No—Race/Ethnicity treated as covariate	No
